# The Impact of Latent *Toxoplasma gondii* Infection on Spontaneous Abortion History and Pregnancy Outcomes: A Large-Scale Study

**DOI:** 10.3390/microorganisms10101944

**Published:** 2022-09-30

**Authors:** Adelina Geanina Mocanu, Dana Liana Stoian, Emanuela Lidia Craciunescu, Ioana Mihaela Ciohat, Alexandru Catalin Motofelea, Dan Bogdan Navolan, Tatjana Vilibic-Cavlek, Vladimir Stevanovic, Dragos Nemescu, Marius Forga, Razvan Daniluc, Alexandra-Magdalena Ioana, Marius Craina

**Affiliations:** 1 Department of Obstetrics-Gynecology, Victor Babes University of Medicine and Pharmacy Timisoara, Eftimie Murgu Sq. no. 2, 300041 Timisoara, Romania; 2 Department of Endocrinology, Victor Babes University of Medicine and Pharmacy Timisoara, Eftimie Murgu Sq. no. 2, 300041 Timisoara, Romania; 3 Department of Prosthesis Technology and Dental Materials, Victor Babes University of Medicine and Pharmacy Timisoara, Eftimie Murgu Sq. no. 2, 300041 Timisoara, Romania; 4 Laboratory of Antenatal Medicine, Timisoara City Emergency Hospital, 300202 Timisoara, Romania; 5 Second Department of Internal Medicine, Victor Babes University of Medicine and Pharmacy Timisoara, Eftimie Murgu Sq. no. 2, 300041 Timisoara, Romania; 6 Department of Virology, Croatian Institute of Public Health, School of Medicine, University of Zagreb, 10000 Zagreb, Croatia; 7 Virology Laboratory, Department of Microbiology and Infectious Diseases with Clinic, Faculty of Veterinary Medicine, University of Zagreb, 10000 Zagreb, Croatia; 8 Department of Obstetrics-Gynecology, Gr. T. Popa University of Medicine and Pharmacy Iasi, Universitatii Str. nr. 16, 700115 Iasi, Romania

**Keywords:** latent toxoplasmosis, spontaneous abortion, pregnancy complications, neonate

## Abstract

Background: *Toxoplasma gondii* (TG), a zoonotic protozoan parasite, belongs to a group of TORCH infectious agents, which can cause severe damage to the fetus if a primary infection occurs during pregnancy. After primary infection, TG rests lifelong in human organisms causing a latent infection. Most studies have analyzed the consequences of acute, but not latent, TG infection. This study analyzed the impact of latent toxoplasmosis on spontaneous abortion history, pregnancy complication rate and neonatal outcome. Methods: IgG and IgM anti-TG antibodies were tested in 806 pregnant women who were consulted at the Timisoara Clinical Emergency Hospital between 2008 and 2010. Demographic data, obstetrical history, and data about the pregnancy complications, birth and neonate were collected for each woman and comparisons between the groups, with and without latent TG infection, were made. Results: This study did not show differences between groups regarding the history of spontaneous abortion (OR = 1.288, *p* = 0.333), cesarean section (OR = 1.021, *p* = 0.884), placental abruption (OR 0.995, *p* = 0.266), pregnancy-induced hypertension rate (OR 1.083, *p* = 0.846), secondary sex ratio (1.043, *p* = 0.776), 1′ APGAR score at birth (*p* = 0.544), gestational age at birth (*p* = 0.491) or birth weight (*p* = 0.257). Conclusions: The observed differences between the rate of pregnancy complications in the two groups of pregnant women with and without latent infection with TG, did not reach a statistical significance.

## 1. Introduction

The birth of a healthy child represents the goal of each obstetrician who cares about a pregnancy [1]. Infectious diseases are events which could cause miscarriage or severe damage to the embryo and fetus under certain conditions [2]. Thus, healthcare policies from some countries (Austria, France, Romania, etc.) include screening tests in routine prenatal care to determine the immunological status of women against certain infectious organisms at the beginning of pregnancy [3,4,5,6]. The rationale for this algorithm is based on the observation that women who have suffered from infectious diseases prior to conception develop specific antibodies, which are a marker of protective immunity against reinfection [7]. Fetuses of these women are mostly not at risk if they are exposed to the same agent. On the other hand, seronegative pregnant women, once infected, will need time to develop antibodies, and this period is sufficient for the pathogenic agent to multiply and pass, via the placenta, to the fetus, causing severe damage [7]. Fetuses of healthy seropositive pregnant women are protected only partially since reinfections, infections by another strain or reactivations of latent infections may occur [8]. Despite the infection of the fetus in these cases, the impact on fetal development is not always severe [8,9]. Women with acquired or genetic immunodeficiency, or who are undergoing immunosuppressive therapy, might also be reinfected [10].

The main pathogens that could cross the placenta following the infection of a pregnant woman and cause severe damage to the offspring belong to the TORCH group, which represents an acronym formed by the initial letters of the names of the following pathogens (*Toxoplasma gondii*, other pathogens, Rubella virus, Cytomegalovirus, Herpes simplex virus) [11]. *Toxoplasma gondii* (TG) is an obligate intracellular zoonotic parasite which can cause several congenital malformations if an acute primary infection occurs during pregnancy [12].

The life cycle of TG includes both sexual and asexual reproduction, the latter taking place in intermediate hosts [13,14,15]. The sexual cycle of TG occurs within the intestines of the Felidae family (definitive hosts), including the domestic cat, after which oocysts are shed in the feces [16,17,18]. Oocysts take one to five days to sporulate in the environment and become infective [19]. Following ingestion by another host, sporulated oocysts transform into tachyzoites, a form of rapidly multiplying cell that is characteristically found in acute infections and can invade almost all vertebrate cell types (neural, muscle, ocular tissue) [15]. Bradyzoites are forms that multiply slowly and are characteristic of chronic infections which originate the tissue cysts [20].

Humans could be infected in several ways: (1) ingestion of water, vegetables or fruits that are contaminated with oocysts sporulated after elimination in cat feces (2) ingestion of undercooked meat that contains viable tissue cysts (bradyzoites) (3) transplacental transmission of tachyzoites (4) blood transfusion containing tachyzoites or (5) organ transplant with tissues contaminated with cysts or tachyzoites [19,20]. The majority of human infections occur by ingestion of undercooked contaminated meat. After ingestion, the bradyzoites invade the intestinal epithelium and differentiate into tachyzoites, which disseminate and replicate within the new host. Host immunity is responsible for controlling parasite replication. The majority of tachyzoites are eventually eliminated by an interferon-gamma (IFN-)-dependent cell-mediated immune response, but in some sites, most frequently the CNS and muscle tissue, including the heart, tachyzoites convert into bradyzoites. Tissue cysts harboring bradyzoites can be detected within six to seven days following infection with oocysts or tissues cysts and are thought to persist throughout the life of the host. However, they are not completely dormant and appear to breakdown occasionally releasing bradyzoites that may convert to tachyzoites, invade adjacent cells, and transform back to bradyzoites, forming new tissue cysts even in immune competent hosts [21].

Humoral immunity is essential for resistance to toxoplasmosis. TG IgM antibodies can be detected approximately seven days after infection and decline within a few months. TG IgG antibodies appear approximately two weeks after infection, peak at three months and persist for life thereafter [22]. Since the acute infection period is the most critical for vertical transmission, research was done to develop immunological tests to identify this period. Thus IgG- and IgM-anti-TG antibody positivity associated with IgA positivity or low IgG avidity is indicative of an acute infection, while IgG-anti-TG positivity, IgM- and IgA-anti-TG negativity or high IgG avidity are indicative of latent infection [23].

In immunocompetent humans, acute toxoplasmosis is usually asymptomatic and pregnant women frequently are unaware that they are infected. Other times, only a few symptoms occur, such as fever, asthenia, headache and swollen lymph nodes [7]. The impact of primary infection at the level of offspring differs according to the gestational age of pregnancy when infection occurs [24]. In early pregnancy, TG infection often causes miscarriage, while in later stages of pregnancy it causes congenital malformations such as intrauterine growth restriction, splenomegaly, hepatomegaly, ocular (chorioretinitis) disturbances or cranial ones (brain calcifications, hydrocephalus, microcephaly and mental retardation) [24]. TG life-long (latent) infection is caused by cysts which are disseminated in various tissues (brain, heart, muscle) and influence the infected host [25,26].

Most studies have analyzed the effect of an acute TG infection on the course of ongoing pregnancy or fetal development, and only a few studies have analyzed the effect of latent toxoplasmosis [2]. Some studies have focused on analyzing the association of latent TG infection with the history of spontaneous abortion [27], the offspring secondary sex ratio [28,29], pregnancy complication prevalence and newborn status [30]. The results of research regarding the impact of latent TG infection on obstetrical history with respect to the above-mentioned pregnancy complications are still controversial and the subject of research [27,28,29,30,31,32,33,34].

Based on our well-structured database, this study aimed to analyze the association between the presence of latent TG infection and demographic features, obstetrical history, pregnancy, and neonatal outcome in 806 pregnant women who were admitted to our clinic.

## 2. Materials and Methods

### 2.1. Study Design, Population, and Setting

A retrospective study was conducted on 806 pregnant women in the western region of Romania from 2008 to 2010 at the Emergency Clinical City Hospital, Timişoara, Romania. Patients were enrolled according to a consecutive case population, based on when they arrived. First-trimester TORCH screening was performed, from 4 to 12 weeks of gestation. IgG-/IgM-anti-TG, anti-CMV, and anti-rubella virus antibodies were determined in all pregnant women. Only pregnant women with negative IgM-anti-TG, -CMV and -rubella virus were included in the study. Pregnant women who had positive IgM-anti-TG, -CMV and -rubella virus titers were further tested with a second serological method, an invasive method in some cases, and they were excluded from the study.

Data about the year of birth, age at blood sampling, and area of residence (urban, rural) were collected at the time of blood sampling for all pregnant women. After birth, retrospective data from medical files referring to the occurrence of pregnancy complications (presence of pregnancy-induced hypertensive disease, premature spontaneous rupture of membranes, placental abruption, abnormal placental insertion, etc.), mode of the establishment of gestational age, gestational age at birth, mode of delivery and data about the newborn (weight at birth, gender, Apgar score, length and head circumference) were identified and added to the data collected at the time of blood sampling. The gestational age was established by ultrasound measurements (first-trimester crown-rump-length or second-trimester composed ultrasound) for all pregnant women. Of the 806 pregnant women with all available data included in the study, 336 women had a cesarean section birth and 470 had a vaginal birth. The effect of latent TG infection on pregnancy duration was examined in 470 pregnant women with vaginal birth.

MoM (multiple of medians) was used to calculate the deviation between the weight at birth and the expected weight of birth for each newborn. Nomograms according to Talge et al. were used to define the expected birth weight of the neonate according to gestational age and gender [35]. For each newborn, an MoM was calculated and the MoMs were compared to analyze the effect of latent TG infection on the newborn’s weight.

### 2.2. Detection of IgG Antibodies against Toxoplasma gondii, CMV and Rubella virus

Measurement of IgG- and IgM-anti-TG, CMV and the anti-rubella-virus antibody titer were performed by a chemiluminescence method, using an Immulite One Machine (DPC, Diagnostic Products Corporation, Los Angeles, CA, USA) and commercial tests (LKTXP1-Immulite *Toxoplasma gondii* quantitative IgG; LKTZ1-Immulite *Toxoplasma gondii* IgM; LKCV1-Immulite CMV IgG; LKCM1-Immulite CMV IgM; LKRUB1-Immulite Rubella IgG quantitatively; LKRM1-Immulite Rubella IgM; Siemens Healthcare Diagnostics Products, Llanberis, Gwynedd, United Kingdom). According to the cut-off values recommended by the manufacturer, patients were grouped into positive, negative, and inconclusive values. For statistical analysis, cases of inconclusive results were considered along with non-immunized cases, due to the uncertainty of immunization.

### 2.3. Statistical Analysis

Astraia database (Astraia GmbH Munich, Ismaning, Germany) and Microsoft Office Excel (One Microsoft Way, Redmond, WA, USA) were used to store data. Instat GraphPad Prism 8.0.2 software (2365 Northside Dr. Suite 560, San Diego, CA, USA) and SPSS version 26 (SPSS Inc, Chicago, IL, USA) were used for statistical analysis. Data are presented as medians and interquartile ranges (IQR) for continuous variables without Gaussian distribution, or percentages for categorical variables. To assess the significance of the differences between groups, Mann–Whitney U test, or Kruskal–Wallis (medians, non-Gaussian populations) and χ^2^ (proportions) tests were used. Continuous-variable distributions were tested for normality using the Shapiro–Wilk test, and for equality of variance using Levene’s test. The strength of association between two continuous variables from non-Gaussian populations was evaluated using the Pearson correlation coefficient. Sample-size calculations were performed before the study, aiming to provide a confidence level of 95%. In this study, *p* < 0.05 was considered the threshold for statistical significance.

The association between the presence of the latent TG infection and the history of spontaneous abortion was analyzed in two ways. First, we counted the number of women with spontaneous abortion (total 84) and compared it with the number of women with at least one previous pregnancy (gestation ≥ 2, total 465) in the group of women with, and, respectively, without latent TG infection. Secondly, we counted the total number of spontaneous abortions (total 108) and compared it with the number of all pregnancies that the women in the two groups had (total 828), with and without TG latent infection.

### 2.4. Ethical Issues

This study was approved by the Institutional Board of the ‘Victor Babes’ University of Medicine and Pharmacy (Timisoara, Romania) (approval no. 848/06.04.2011). The study meets the ethical guidelines, including adherence to the legal requirements of the study country.

## 3. Results

### 3.1. Demographic and Clinical Features of Pregnant Women Included in the Study

Of the pregnant women in this study, 72.6% came from urban areas, 11.4% were smokers; the women had a median age of 28 years and a median body mass index (BMI) of 21.8 kg/m^2^ at the beginning of the pregnancy, which is within the normal range. Of 806 pregnant women, 42.6% (*n* = 343) had a latent TG infection (IgG seropositive). The main parameters related to pregnancy and neonatal outcomes are presented in Table 1.

### 3.2. Latent *Toxoplasma gondii* Infection and the Rate of Previous Spontaneous Abortion

No association was found between the presence of latent TG infection and the history of spontaneous abortion regardless of our counting (A) the number of women with spontaneous abortion (total 84) and comparing it with the number of women with at least one previous pregnancy (gestation ≥ 2, total 465), or counting (B) the total number of spontaneous abortions (total 108) and comparing it with the number of all pregnancies the women in the two groups had (total 828), with and without TG latent infection (Table 2).

### 3.3. Latent *Toxoplasma gondii* Infection and the Course of Pregnancy with Respect to the Neonatal Outcome

Our results did not find any association between the presence of latent TG infection and fetal presentation, mode of delivery, the prevalence of spontaneous rupture of membranes, abnormal placenta insertion, placental abruption, or pregnancy-induced hypertensive disease. No association was found between the status of latent TG infection and the recorded neonatal features: secondary sex ratio, 1′ and 5′ APGAR scores, birth weight, gestational age at birth, length, or head circumference (Table 3). Also, no difference was found regarding gestational age at birth expressed in days, birth weight expressed in multiple to median (MoM) or length and head circumference between the two groups—with, versus without latent TG infection.

## 4. Discussion

To our knowledge, this study is, worldwide, one of the few studies to analyze the impact of latent TG infection on the history of spontaneous abortion [18,22,23,24,25,26], secondary sex ratio [28,36,37,38,39], occurrences of pregnancy complications (such as the mode of delivery), the prevalence of spontaneous premature rupture of membranes, pregnancy-induced hypertensive disease [30,40,41,42], preterm birth or prolonged pregnancy [12,43], with respect to neonatal outcome. The gestational age was established by the first or second-trimester scan in each patient. This confers high accuracy, allowing accurate comparison of the birth weight of each neonate to controls considering the gestational age and gender.

TG, after initial infection, modulates the immune system and develops a latent infection. Several reports suggest that latent infection may be responsible for development of several clinical entities and diseases of the host, as described by Flegr elsewhere [14,26]. Depending on the strain involved in the infection, toxoplasmosis varies in clinical presentation from asymptomatic to severe forms, and it influences the immune system of the host in multiple ways, for example, using dendritic cells as a Trojan Horse for dissemination [44,45] or by peripheral blood cytokine regulation [46]. Previous studies have shown that TG elicits multiple effects on human hosts [14]. It was reported that latent toxoplasmosis was associated with obesity [47], thyroid dysfunction in the general population [48], the presence of anti-thyroid antibodies in pregnant women [49], arterial hypertensive disorders in type-2 diabetes mellitus patients [50] and psychiatric disorders [26,51,52].

Previous studies have suggested that latent TG infection affects the course of pregnancy, thus seropositive pregnant women show a modified secondary sex ratio in newborns [28,29,37,38,39], more spontaneous abortion [27,31,33,36], more prolonged pregnancies [43], obesity [47] or higher weight gain in pregnancy in Rh-negative women, higher pregnancy-induced hypertension disease rate [40,50] and higher prevalence of autoimmune thyroiditis [28,48,49]. In addition, previous publications mentioned that acute TG infection during pregnancy is associated with miscarriage [27] or congenital malformations in fetuses [2] and reactivation of latent infection or infection with another strain could also be a cause of congenital malformations [6,7].

Studies from Iran [27,31], Mexico [32], and Yemen [33] analyzed the association between TG seroprevalence and the history of spontaneous abortion and found controversial results. While the study from Durango City, Mexico, performed on 326 women with a history of miscarriage, did not find an association between a history of miscarriage and TG seroprevalence [32], the study from Yemen, which included 420 pregnant women, showed a significant association between TG seroprevalence and the history of spontaneous abortion [33]. The meta-analysis by Kalantari et al. from Iran included 40 studies, of which 17 reported a significant association, whereas the other 23 studies concluded against the presence of an association between spontaneous abortion rate and latent TG infection [31]. Conversely, another meta-analysis from Iran, performed by Nayeri et al., suggests a possible relationship between latent toxoplasmosis and miscarriage history [27]. Our study did not report an association between the presence of latent TG infection and a history of miscarriage, whether the cases were counted as patients or as all pregnancies to calculate the odds ratio.

Some published studies have analyzed the association between latent TG infection and the sex ratio at birth and concluded that women infected with TG have more sons [28,29,36,37,38,39]. Many hypotheses have been raised to explain this association: modulation of the immune response and cytokine production [36] or increased testosterone levels in TG seropositive women [39]. A more detailed analysis performed on mice by Kankova et al. showed that TG-infected mice showed a higher male-to-female newborn sex ratio than controls in the early period of the latent infection, whereas in the late period of the latent infection, the sex ratio decreased [38]. Our results showed a slightly increased male-to-female sex ratio in newborns of women with latent TG infection, but the difference did not reach a significant threshold (OR = 1.043, *p* = 0.776).

Also, our study did not find an association between TG seroprevalence and abnormal fetal presentation at delivery, cesarean section rate, abnormal placenta insertion, placental abruption, or pregnancy-induced arterial hypertension prevalence.

Special attention was given to the study of the association between pregnancy-induced hypertensive disease prevalence and latent TG infection [30,40,41,42]. Thus, some researchers have raised the hypothesis that preeclampsia could be an infectious disease and shown that patients, which are seronegative to CMV and HSV have a higher prevalence of preeclampsia [41]. Patients who were seronegative to TG did not show such a pattern [41]. Furthermore, a study from North Mexico did not find an association between seroprevalence to TG and hypertensive disorders in pregnancy [42]. On the other hand, a study from Italy showed that women treated with spiramycin for toxoplasmosis had a lower incidence of hypertensive disorders in pregnancy than untreated ones [40]. The results of our study did not show an association between latent TG infection and pregnancy-induced hypertension prevalence.

Although some studies showed that latent TG infection could cause prolonged pregnancies [43], our results did not confirm this hypothesis. No differences were found between gestational age at birth between pregnancies with or without latent TG infection whether the analysis was done in the entire group of pregnant women or stratified according to gestational age subcategories [53].

A recent study from the Czech Republic (2022) by Hurt et al., performed on 1733 pregnant women, found a higher prevalence of preterm birth and low birth weight fetuses in pregnant women with latent TG infection [54], but a study from Brazil (2015), performed on 213 pregnant women, showed that prematurity and low birth weight did not correlate with anti-TG maternal serum profiles [12]. Our results did not show a higher preterm birth or low birth weight prevalence in pregnant women with latent toxoplasmosis.

In addition, our study did not find a difference between APGAR Score (1 min or 5 min), length or head circumference of neonates related to the presence of latent TG infection.

The strength of our study is that it is based on a well-characterized database in which all patients had gestational age established to an accuracy of a maximum of 5 days according to the first or second-trimester scan [55]; this allows us to accurately assess the prevalence of pregnancy complications, for which exact dating is essential, such as determining the degrees of prematurity or comparing the weight of the newborns in the study with the ideal weight of the newborns, according to gender and gestational age at birth. It is also one of the few articles that analyzes the impact of latent infection not only on one complication but on several pregnancy complications at the same time.

Our study had also some limitations. The main limitation is that the study was conducted in a single hospital which covers a region of only three counties (Timis, Arad, Caras-Severin) situated in the western part of Romania—a region with a population of around 1,000,000 residents using our maternity services. In addition, the study reflects only the impact of the latent infection caused by the TG strain that is widespread in our region. To our knowledge, TG strain 2 is predominantly found in our region, a strain with less severe pathogenicity compared to the other strains [56]. This could be the explanation for the lack of a statistically significant difference in the rate of pregnancy complications between the two groups of pregnant women—with or without latent TG infection. Another limitation of this study is that we had no opportunity to analyze the rate of reinfection in pregnant women with latent infection or to analyze whether the history of spontaneous abortion or the prevalence of pregnancy and neonatal complications are correlated with the IgG-anti-TG titers. Further studies are necessary to clarify these questions.

## 5. Conclusions

In conclusion, in our study, the differences between the rate of pregnancy complications in the two groups of pregnant women, with and without latent infection with TG, did not reach statistical significance. Several factors, such as conducting the study in a single center and region or analyzing the impact of the latent infection caused by a single, less pathogenic strain on pregnancy complications, could have contributed to these results. The accuracy of the characterization of pregnant women, especially regarding the complications that require an exact dating, makes our results valid and useful to include in meta-analyses that will accumulate a larger number of cases and provide stronger statistics.

## Figures and Tables

**Table 1:** Pregnant women’s baseline features: demographic, obstetrical history, pregnancy and neonatal outcome.

Pregnant Women (*n* = 806)		N (%), Median [IQR]			N (%), Median [IQR]
			Pregnancy outcome		
Age of women (years) ^b^		28 [25–31]	Birth mode ^a^	Vaginal	470 (58.3%)
Provenance ^a^	Rural	219 (27.2%)		Cesarean	336 (41.7%)
	Urban	585 (72.6%)	Spontaneous rupture of membrane ^a^	No	601 (74.8%)
	Unknown	2 (0.2%)		Yes	202 (25.2%)
Smoker ^a^	No	673 (83.5%)	Placenta insertion ^a^	Normal	799 (99.1%)
	Yes	92 (11.4%)		Previa	7 (0.9%)
	Unknown	28 (3.5%)	Placental abruption ^a^	No	803 (99.6%)
	Stopped	13 (1.6%)		Yes	3 (0.4%)
Height (cm) ^b^		165 [160–168]	PIH ^a^	No	775 (96.6%)
Weight—at onset of pregnancy (kg) ^b^		59 [53–67]		Yes	27 (3.3%)
BMI—at onset of pregnancy (g/m^2^) ^b^		21.8 [19.7–25.0]	Neonatal outcome		
Gestation ^a^	1	341 (42.3%)	Gender ^a^	Girls	402 (49.9%)
	2	261 (32.4%)		Boys	404 (50.1%)
	3	115 (14.3%)	APGAR 1’ ^a^	<7	23 (2.9%)
	4	55 (6.8%)		8–9	658 (81.6%)
	5	18 (2.2%)		10	61 (7.6%)
	6	7 (0.9%)		Unknown	52 (7.9%)
	7	5 (0.6%)	APGAR 5’ ^a^	<7	6 (0.7%)
	8	2 (0.2%)		8–9	650 (80.6%)
	10	1 (0.1%)		10	98 (12.2%)
	13	1 (0.1%)		Unknown	52 (6.5%)
History of spontaneous abortion ^a^	0	722 (89.6%)	Birthweight (g) ^a^	<1500	6 (0.7%)
	1	62 (7.7%)		1500–2500	39 (4.8%)
	2	20 (2.49%)		2500–4000	723 (89.7%)
	3	2 (0.25%)		>4000	38 (4.7%)
Previous cesarean ^a^	No	742 (92.1%)	GA (weeks) ^a^	<28	0 (0.0%)
IgG anti-TG ^a^	Yes	64 (7.9%)		29–32	7 (0.9%)
	Negative	463 (57.4%)		33–34	11 (1.4%)
	Positive	343 (42.6%)		35–37	57 (7.1%)
Pregnancy outcome				37–43	731 (90.7%)
Presentation at delivery ^a^	Cephalic	756 (93.8%)	GA (days) ^b^	273 [267–278]	
	Breech	50 (6.2%)	Birthweight (MoM) ^b^	0.998 [0.923–1.017]	

^a^ Categorical variables are represented as counts and percentages. ^b^ Numeric variables are represented as median and interquartile range [IQR]. PIH—pregnancy-induced hypertension, BMI—body mass index, GA—gestational age, MoM—multiple of median, TG—*Toxoplasma gondii*.

**Table 2:** Association between latent *Toxoplasma gondii* infection and history of spontaneous abortion.

		TG IgG Antibodies				OR [CI]	*p* Value
		Positive		Negative			
		Number	N%	Number	N%		
Obstetrical history							
A—counted as persons (*n* = 465)	Pregnancies	171	79.9%	210	83.6%	1.288 [0.902–2.067]	0.333
	SA	43	20.1%	41	16.3%		
B—counted as pregnancies (*n* = 828)	Pregnancies	354	86.1%	366	87.7%	1.156 [0.770–1.733]	0.536
	SA	57	13.8%	51	12.2%		

SA—spontaneous abortion, OR—odds ratio, CI—confidence interval.

**Table 3:** Association between latent *Toxoplasma gondii* infection with pregnancy complications (a) and neonatal features (b).

		TG IgG Antibodies				OR [CI]	*p* Value
		Negative		Positive			
		Count	N%	Count	N%		
a. Pregnancy complications							
Fetal presentation	Cephalic	429	92.7%	327	95.3%	0.617 [0.335–1.138]	0.078
	Non-cephalic	34	7.3%	16	4.7%		
Mode of delivery	Vaginal	271	58.5%	199	58%	1.021 [0.770–1.356]	0.884
	Cesarean	192	41.5%	144	42%		
Spontaneous rupture of membranes	No	347	74.9%	254	74.1%	1.013 [0.734–1.398]	0.935
	Yes	116	25.1%	86	25.9%		
Placental insertion	Normal	460	99.4%	339	98.8%	1.809 [0.402–8.137]	0.467
	Praevia	3	0.6%	4	1.2%		
Placental abruption	No	460	99.4%	343	100%	0.994 [0.986–1.001]	0.266
	Yes	3	0.6%	0	0%		
Pregnancy-induced hypertension	No	448	96.8%	331	96.5%	1.083 [0.500–2.344]	0.846
	Yes	15	3.2%	12	3.5%		
b. Neonatal features							
Secondary sex ratio	Girls	233	50.3%	169	49.3%	1.043 [0.789–1.379]	0.776
	Boys	230	49.7%	174	50.7%		
APGAR 1′	<7	12	2.8%	11	3.5%	1.660 [0.627–4.40]	0.544
	8–9	380	88.2%	278	89.4%	1.285 [0.743–2.20]	
	10	39	9%	22	7.1%	R	
APGAR 5′	<7	3	0.7%	3	0.9%	1.551 [0.228–7.93]	0.881
	8–9	373	85.9%	277	86.6%	1.079 [0.700–1.66]	
	10	58	13.4%	40	12.5%	R	
Birth weight categories (grams)	≤1500	5	1.1%	1	0.3%	0.282 [0.032–2.436]	0.350
	1501–2500	22	4.8%	17	5.0%	1.063 [0.554–2.039]	
	2501–4000	418	90.3%	305	88.9%	R	
	>4000	18	3.9%	20	5.8%	1.526 [0.793–2.936]	
Gestational age at birth categories (weeks)	<32	6	1.3%	1	0.3%	0.221 [0.026–1.848]	0.434
	32–33	5	1.1%	6	1.7%	1.591 [0.480–5.267]	
	34–36	36	7.8%	21	6.1%	0.773 [0.440–1.358]	
	37–39	325	70.2%	245	71.4%	R	
	≥40	91	19.7%	70	20.4%	1.020 [0.716–1.452]	
		Median [IQR]		Median [IQR]			
Gestational age (days)		273 * [266–278]		273 * [267–279]			0.491
Birth weight (MoM)		0.99 * [0.922–1.067]		1.01 * [0.929–1.089]			0.257
Length (cm)		50 * [49–52]		50 * [49–52]			0.232
Head circumference (cm)		34 * [33–35]		34 * [33–35]			0.973

* Median [IQR], R—Reference, OR—Odds Ratio.

## Data Availability

The data sets used and/or analyzed during the present study are available from the first correspondence author upon reasonable request.
